# Hybrid UV/COP advanced oxidation process using ZnO as a catalyst immobilized on a stone surface for degradation of acid red 18 dye

**DOI:** 10.1016/j.mex.2020.101118

**Published:** 2020-10-29

**Authors:** Hakimeh Mahdizadeh, Alireza Nasiri, Majid Amiri Gharaghani, Ghazal Yazdanpanah

**Affiliations:** a Environmental Health Engineering Research Center, Kerman University of Medical Sciences, Kerman, Iran; b Department of Environmental Health, School of Public Health, Kerman University of Medical Sciences, Kerman, Iran; c Department of Environmental Health Engineering, Sirjan School of Medical Sciences, Sirjan, Iran

**Keywords:** AOP process, Dyes, ZnO, Water treatment, Textile industry

## Abstract

Azo dyes are the largest group of synthetic organic dyes which containing the linkage C—N=N—C and used in various industries such as textile industries leather articles, and some foods. Azo dyes are resistant compounds against the biodegradation processes. The purpose of this research was hybrid UV/COP advanced oxidation process using ZnO as a catalyst immobilized on a stone surface for degradation of acid red 18 (AR18) Dye. In the hybrid process using some parameters such as the dye initial concentration, pH, contact time and catalyst concentration, the process efficiency was investigated. In order to the dye removal, the sole ozonation process (SOP), catalytic ozonation process (COP) and photocatalytic process (UV/ZnO) were used. The ZnO nanoparticles were characterized by XRD, SEM and TEM analyses.  The maximum dye removal was achieved 97% at the dye initial concentration 25 mg/L, catalyst concentration 3 g/L, contact time 40 min and pH 5. As a real sample, the Yazdbaf textile factory wastewater was selected. After that, the physicochemical quality was evaluated. As well as, in the optimal conditions, the AR18 dye removal efficiency was achieved 65%. The kinetic results demonstrated that the degradation reaction was fitted by *pseudo*-first-order kinetic. The UV/COP hybrid process had high efficiency for removal of resistant dyes from the textile wastewater.

Advantages of this technique were as follows:

• ZnO nanoparticles were synthesized as catalyst by thermal method and were immobilized on the stones.
• pH changes had no significant effect on the removal efficiency.
• In the kinetic studies, the decomposition reaction followed *pseudo*-first order kinetic.

## Graphical abstract

**Figure:**
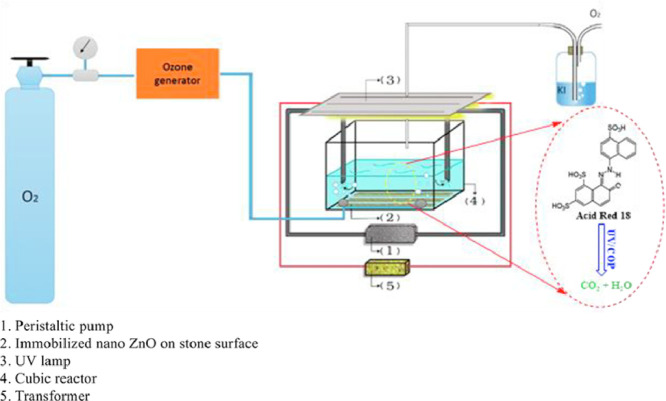

Specifications table

**Table:** 

Subject Area:	• Environmental Sciences
More specific subject area:	*Chemical engineering in environmental sciences*
Method name:	*Hybrid UV/COP advanced oxidation process using ZnO as a immobilized catalyst on stone surface for degradation of* AR18
Name and reference of original method	*Malakootian M, Smith Jr. A, Amiri Gharaghanid M, Mahdizadehb H, Alireza Nasiri A, Yazdanpanah G. Decoloration of textile AR18 dye by hybrid UV/COP advanced oxidation process using ZnO as a catalyst immobilized on a stone surface. Desalination and Water Treatment. (2020); 182: 385–394.* DOI:10.5004/dwt.2020.25216
Resource availability	

## Introduction

The textile industry wastewater contains many contaminants such as dyestuff, chemical compounds and nonionic surfactants therefore, the textile industry is one of the most polluting industries [1]. Azo dyes are non-degradable, resistant to light, have one or more nitrogen-nitrogen band. These dyes are inexpensive and have high solubility and stability [2]). Due to azo dyes variety structures and easy to make are the most used among dye compounds [3]. The azo days could cause some environmental and health problems such as cancer, renal and liver dysfunctions [4]. Hence, removal of azo dyes from textile industry wastewater is so necessary. Lately, a lot of physical, chemical and biological methods [5,6] such as adsorption [7], [8], [9], [10], [11], [12], [13], electrocoagulation [14], [15], [16], [17], activated sludge [18], photochemical [19], [20], [21], [22], [23], [24], [25], [26], [27], [28], [29], [30], [31]), oxidation [30], [31], [32], [33], [34], trickling filter [35] and membrane processes [36] have used for the azo dyes, organic and inorganic pollutants removal from the environment. However, these methods have some problems [23] such as the toxics production expensive facilities and high maintenance and operation costs.

The catalytic ozonation method is an effective process for the azo dyes and other pigments removal compared to the other processes [37], [38], [39], [40]. Ozone is a powerful oxidant and dissolved in water and uses in the waters and wastewaters treatment [41]. In addition, single ozone doses could therapeutically use in the selected human diseases. Although, conventional ozonation technologies have some limitations such as low ozone efficiency ozone poor mass transfer from the gas phase to liquid phase and the limited organics mineralization [42,43]. To solve some of these problems, use of a catalyst has been suggested. Using catalyst could increase the oxidants generation such as hydroxyl radicals and as a result the process efficiency will enhance [44,45].

In the catalytic ozonation systems, the nature of generated oxidants depend on the catalyst used type [46]. For example, interaction between ozone and CaO_2_ could generate superoxide which enhance the ozone transformation to OH^−^ [47]. Some researchers reported that the surface atomic oxygen formation will happen in the presence of catalyst when single oxygen and HO_2_^−^ be dominant oxidants [48].

In the literature NiFe_2_O_4_ [49], Fe, Cu, Ru, and Ag precursors to Mn/HZSM-5 [50], nano-Fe_3_O_4_@cow dung ash [51], MgO [52], Fe_3_O_4_/MnO_2_ [53], Ce/Al_2_O_3_ [54], graphite felt supported MgO [55] and MgO/AC [56] as heterogeneous catalysts were used in the presence of O_3_ to produce ^•^OH to increase organic oxidation.

Due to, previous studies literature review, there is no research about the AR18 removal using hybrid UV/COP advanced oxidation process using ZnO as a new method for degradation of AR18 dye from aqueous solutions.

## Method

The study steps were as follows: first of all, the ZnO nano particles were prepared with thermal method and were characterized by using SEM, XRD and TEM analysis. Then, in order to process operation, a batch reactor was designed. After that, the photolysis, sole ozonation, catalytic ozonation and photocatalytic processes were compared with each other. In the next step, the operational parameters which affected on the AR18 removal efficiency were evaluated and optimized. At next level, the physicochemical quality of Yazdbaf textile factory wastewater was determined. The AR18 degradation kinetic with hybrid UV/COP advanced oxidation process was investigated. Finally, in the optimal conditions, the AR18 degradation from wastewater was evaluated.

## Chemicals

CH_3_CH_2_OH, H_2_SO_4_, NaOH, ZnSO_4_, 7H_2_O and Na_2_CO_3_ were purchased from Merck Company (Germany) and used without more purification. AR18 dye (99% purification) was obtained from Alvan Paint Company (Tehran, Iran). In addition, suitable dimension waste stones with flat surfaces were used as catalyst bed. All the materials were in analytical grade and used without further purification. Deionized water (DW) was used during the tests.

## ZnO nanoparticles synthesis and characterization

**Fig. 1:**
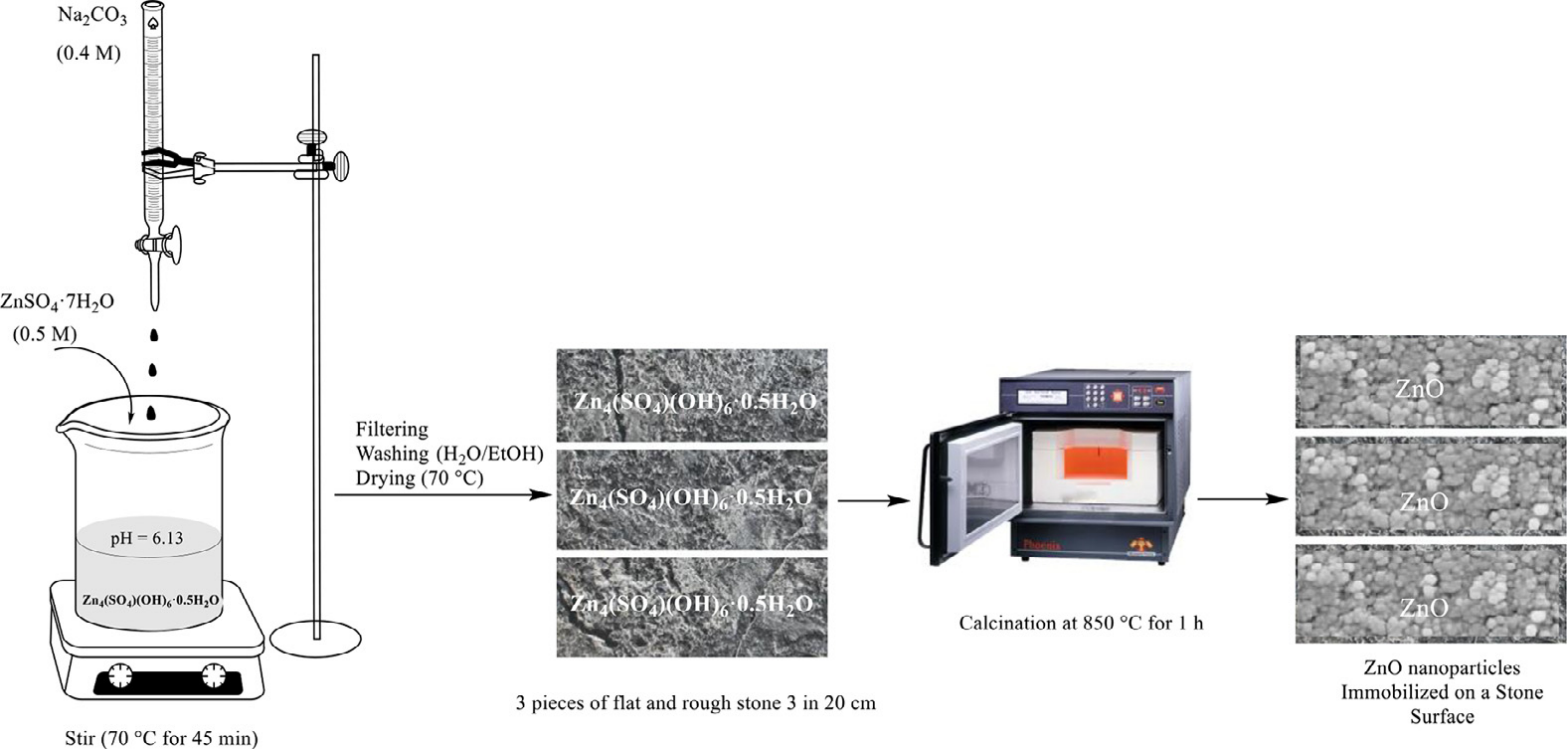
Experimental procedure for the synthesis of nano-ZnO and immobilization on the stone surface.

By thermal method according to Zn_4_(SO_4_)(OH)_6_.0·5H_2_O conversion, ZnO nanoparticles were synthesized. First of all, a 0.5 molar solution of ZnSO_4_·7H_2_O and 0.4 molar solution of Na₂CO₃ were prepared. In the next step, Na₂CO₃ solution was added gradually to ZnSO₄ solution in order to form Zn_4_(SO_4_)(OH)_6_.0·5H_2_O with high speed mixing, at 70 °C for 45 min. After that the obtained sediment was collected and washed with ethanol and DW then dried at 70 °C in the oven. Eventually, the precursor was immobilized on 3 pieces of flat and rough stone 3 in 20 cm and in order to calcination it was placed in an electric furnace (BADi) for 1 hour at 850 °C (Fig. 1) [19].

In order to measure the output and initial dye concentrations a UV spectrophotometer (Shimadzu model, Japan) at a wavelength of 507 nm was used. To determine the constituent phases and the ZnO nanoparticles crystallite size that immobilized on the stone surface, an X-ray diffraction analyzer (XRD) (Philips X'PERT) was used. The microscopic structure, morphology and the size of synthesized ZnO nanoparticles were determined by using a scanning electron microscope (SEM) (KYKY-EM3200, China) and transmission electron microscope (TEM: Philips CM30, Netherland). The reaction kinetic was studied with the *pseudo*-first order equation. The obtained results were analyzed using SPSS-22 software.

## Reactor design and the process operation

**Fig. 2:**
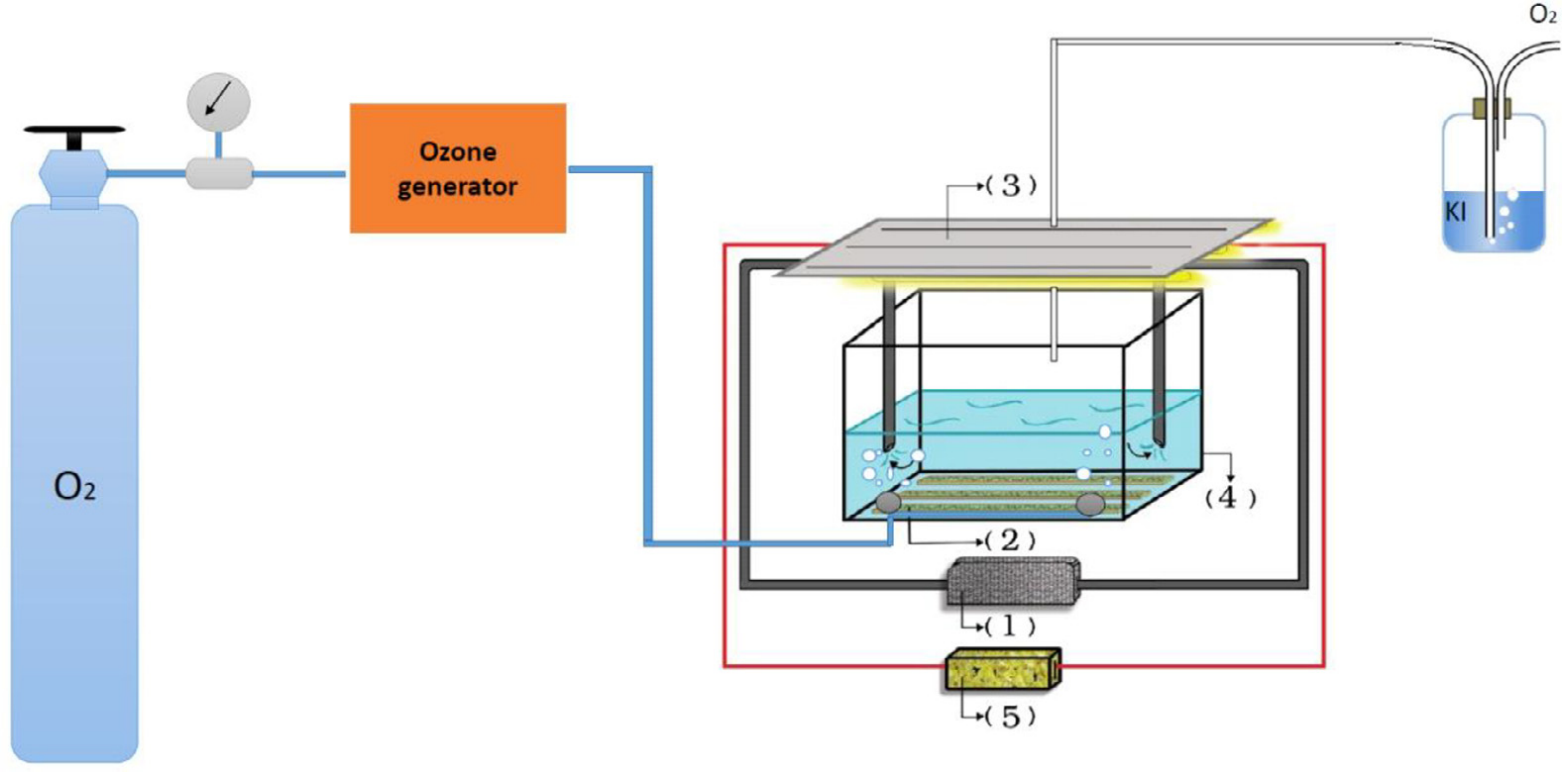
Peristaltic pump [1], ZnO synthesized nanoparticles on stone bed [2], UV lamp [3], cubic reactor [4], Transformer [5].

A plexiglass laboratory-scale reactor with dimensions of 10 × 25 × 5 cm, as shown in Fig. 2, was used to do the experiments. On the top of the reactor, three UV-C lamps (6-watt) were placed. To produce ozone, ozone generators (Modular Ozone Generator, France) were used. The ozone was injected into the reactor. To produce hydroxyl radicals, the distance between the UV irradiation source and the catalyst bed surface, was set about at 2 cm. Materials were mixed using a peristaltic pump with a flow rate of 1 mL/s the reactor.

AR18 synthetic solution was prepared and stored in a dark place. 350 mL of solution was poured inside the reactor in the presence of UV lamps and ozone. After that, the effects of various dye concentrations (25, 50, 75, 100 mg/L), different pH's [4,^7^,^9^,11], and different times in removal of the dye efficiency (5, 10, 15, 20, 25, 30, 35, 40 min) were investigated. The various processes effects such as SOP, COP, photolysis and photocatalysis were investigated. In the optimum conditions, the amount of dye removal was investigated and also, the physicochemical quality of Yazdbaf textile factory wastewater was evaluated. Because of low concentration of AR18 dye in wastewater, this dye was added to the wastewater sample until the dye concentration reaches to 25 mg/L. In this study, the total sample size was 128. All experiments were repeated triplicate. The results were reported as mean with standard deviations.

## Comparison of the SOP, photocatalysis (ZnO/UV), photolysis (UV) and COP processes

**Fig. 3:**
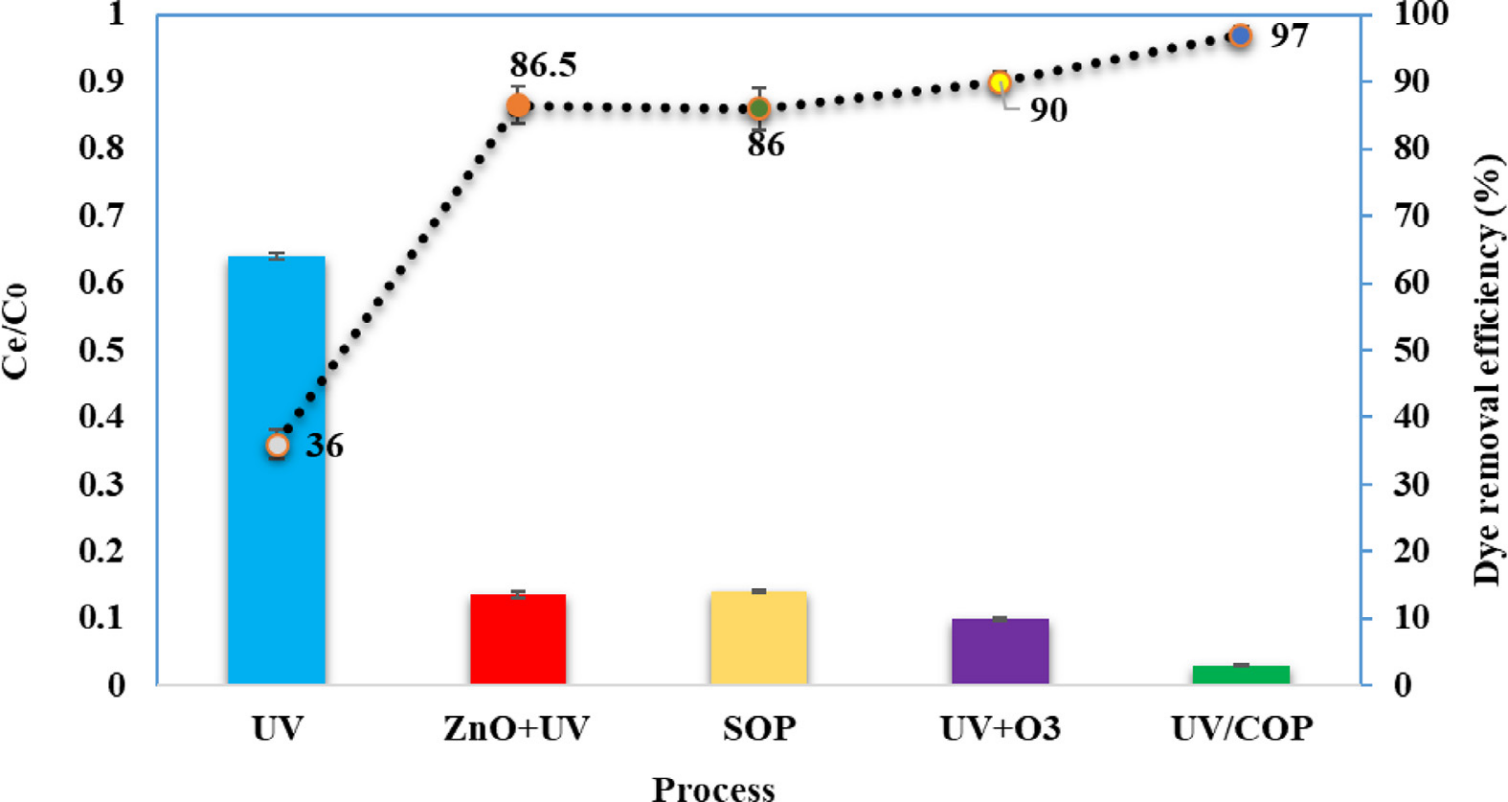
The involved processes effect in the AR18 removal rate with a hybrid UV/COP process (dye initial concentration: 25 mg/L, contact time: 40 min, pH: 6.5).

The AR18 removal efficiency in the SOP, photocatalysis (ZnO/UV), photolysis (UV) and COP processes were compared. In the same conditions, removal efficiency of dye for each process was tested and by comparison the obtained results which shown in Fig. 3, the effect of each process was determined.

As shown in Fig. 3, in the AR18 initial concentration of 25 mg/L, contact time 40 min and at pH 6.5 in the photolysis (UV), sole ozonation process (SOP), ozonation under UV irradiation, UV/COP ozonation and photocatalysis (ZnO/UV) processes, the rate of dye removal was achieved at 36, 86.5, 86, 90, and 97%, respectively.

## Operational parameters optimization on the decomposition of AR18

Effects of the dye various concentrations, different pHs, and different times in the dye removal efficiency were optimized. The maximum dye removal efficiency, by using the UV/COP hybrid process in the dye initial concentration 25 mg/L, pH 5, contact time 40 min and catalyst concentration 3 g/L was achieved 97%. The hybrid process had high efficiency in all pH ranges. pH change did not produce significant change in the efficiency process. In the acidic and basic conditions, the hybrid process was able to remove the resistant organic compounds.

## The kinetic study of AR18 degradation

To describe the dye removal, *pseud*o-first order kinetic is used which is commonly used for degradation of azo dyes in the advanced oxidation processes and the dye removal in the liquid leachate process*.* Therefore, based on the Eq. (1) and by using the *pseud*o-first order kinetic model, the AR18 degradation kinetic [57] in the range of 0–40 min and at concentrations 25, 50, 75 and 100 mg/L and was evaluated. After that, in order to calculate the required time to achieve the 99% dye removal rate, Eq. (2) was used [58].

(1) $$Ln\left(C_{0}/C_{t}\right)=-K_{1}t$$

(2) $$t_{99}=-Ln\;0.01/K$$

where *K*_1_ is the *pseudo*-first order kinetic equation rate constant (1/min), *C*_0_ is the equilibrium concentration and *C_t_* is the concentration at time “*t*” [59].

**Fig. 4:**
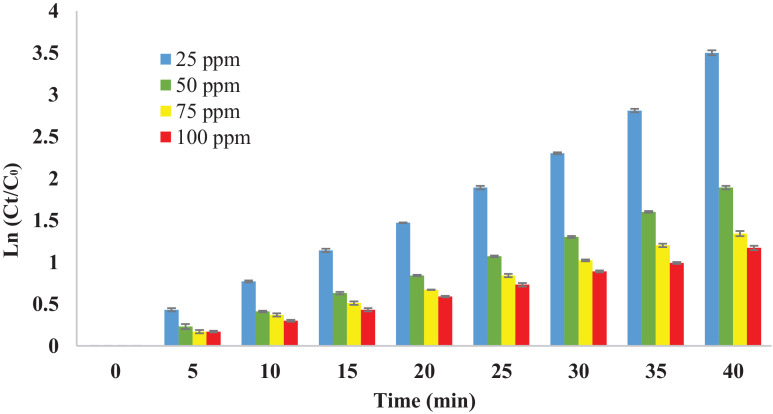
The AR18 degradation kinetic in the hybrid UV/COP process (pH: 5, catalyst concentration 3 g/L, O_3_: 2.55 mg/min).

The *pseudo*-first order kinetic diagram of dye degradation in the hybrid UV/COP process has been shown in Fig. 4. The rate constant was calculated with the linear graph, which was obtained by drawing Ln (C_0_/C_t_) in contrast to the reaction time.

In Table 1 the rate constant (*K*), correlation coefficient (*R*^2^), and time required to degrade 99% of the AR18 dye are shown.

**Table 1:** The results of the *pseudo*-first order kinetic model for dye degradation in the hybrid UV/COP process.

C_0_ (mg/L)	R^2^	K (1/min)	t_99_ (min)
25	0.9887	0.083	55.5
50	0.9947	0.046	100
75	0.9992	0.033	139.5
100	0.9987	0.028	164.5

According to the obtained results which shown in Table 1, in the hybrid UV/COP process, between 56 and 165 min, the AR18 dye removal rate was achieved 99%. The correlation coefficient (R^2^) of more than 99% in the *pseudo-*first order kinetic equation for the different dye concentrations approves that the *pseud*o-first order kinetic model is appropriate for describing the AR18 dye removal efficiency. As well as, by increasing the color concentration, the rate constant speed decreases [58,60].

## Yazdbaf textile factory wastewater physicochemical quality and AR18 degradation from wastewater

The physicochemical quality of Yazdbaf textile factory wastewater was achieved pH: 8.9, EC: 4850 µs/cm, Turbidity: 660 NTU, COD: 3200 mg/L, BOD_5_: 350 mg/L, TDS: 9000 mg/L, TSS: 2800 mg/L, detergent: 120 mg/L, benzene: 145 mg/L, Cr: 18 mg/L, Pb: 30 mg/L, Ni: 14 mg/L, respectively. The removal efficiency percentage of AR18 dye from Yazdbaf textile factory wastewater, in the optimum conditions was obtained 65%. In the real sample, the effect of pollutant influence was investigated. In the both synthetic and real sample, the dye removal efficiency decreased from 97% to 65%. In the hybrid UV/COP advanced oxidation process, the organic compounds degradation happen via two mechanisms types in the presence of ozone. Ozone directly reacts with compounds which have double bonds and also, organic matters such as detergents and benzene in the wastewater. However, in order to produce the hydroxyl radicals, UV/O_3_ reacts non-selectively with all compounds in the wastewater, which leads to reduce the removal efficiency of dye molecules. Free radicals that generated from ozone could indirectly react with organic matters. Dye molecules degraded with ozone and hydroxyl radicals in synthetic sample and produce intermediates.

## Conclusion

In summaries, the UV/COP hybrid oxidation was an efficient process in the removal of AR18 dye from textile wastewater. The UV/COP hybrid oxidation process was the most efficient method in the dye removal compared to the SOP, COP, and photocatalysis processes. A maximum dye removal efficiency using the UV/COP hybrid process was obtained 97% at contact time 40 min and the initial dye concentration 25 mg/L. The hybrid process showed high efficiency in all pH ranges. pH change did not make a significant change in the efficiency process. Considering that the UV/COP hybrid oxidation process could remove resistant organic compounds in the both acidic and basic conditions, this method can be applied for removal of some environmental pollutants in the different industries.

## Declaration of Competing Interest

The authors declare that they have no competing financial interests.

## References

[1] Mortadi A., Chahid E.G., Elmelouky A., Chahbi M., El Ghyati N., Zaim S. (2020). Complex electrical conductivity as a new technique to monitor the coagulation-flocculation processes in the wastewater treatment of the textile Industry. Water Resour. Ind..

[2] Xie Y., Xie S., Yang H., Deng Y., Qian H., Zeng X (2020). A dramatically improved degradation efficiency of azo dyes by zero valent iron powders decorated with in-situ grown nanoscale Fe2B. J. Alloys Compd..

[3] Dheyab M.A., Owaid M.N., Rabeea M.A., Aziz A.A., Jameel M.S (2020). Mycosynthesis of gold nanoparticles by the Portabello mushroom extract, Agaricaceae, and their efficacy for decolorization of Azo dye. Environ. Nanotechnol. Monit Manag..

[4] Al-Tohamy R., Sun J., Fareed M.F., Kenawy E.-.R., Ali S.S (2020). Ecofriendly biodegradation of Reactive Black 5 by newly isolated Sterigmatomyces halophilus SSA1575, valued for textile azo dye wastewater processing and detoxification. Sci. Rep..

[5] Kingston Stanley P., Sanjeevi Gandhi A., Suresh Kumar K., Kalidass S (2019). Process optimization of color removal from an industrial azo dye. Int. J. Innov. Technol. Explor. Eng..

[6] Khosravi A., Javdan M., Yazdanpanah G., Malakootian M (2020). Removal of heavy metals by Escherichia coli (E. coli) biofilm placed on zeolite from aqueous solutions (case study: the wastewater of Kerman Bahonar Copper Complex). Appl. Water Sci..

[7] Ali F., Ali N., Bibi I., Said A., Nawaz S., Ali Z. (2020). Adsorption isotherm, kinetics and thermodynamic of acid blue and basic blue dyes onto activated charcoal. Case Stud. Chem. Environ. Eng..

[8] Javid N., Nasiri A., Malakootian M (2019). Removal of nonylphenol from aqueous solutions using carbonized date pits modified with ZnO nanoparticles. Desalin. Water Treat..

[9] Malakootian M., Nasiri A., Mahdizadeh H (2019). Metronidazole adsorption on CoFe2O4 /activated carbon@chitosan as a new magnetic biocomposite: modelling, analysis, and optimization by response surface methodology. Desalin. Water Treat..

[10] Malakootian M., Nasiri A., Mahdizadeh H (2018). Preparation of CoFe 2 O 4 /activated carbon@chitosan as a new magnetic nanobiocomposite for adsorption of ciprofloxacin in aqueous solutions. Water Sci. Technol..

[11] Malakootian M., Hashemi M., Toolabi A., Nasiri A (2018). Investigation of nickel removal using poly(amidoamine) generation 4 dendrimer (PAMAM G4) from aqueous solutions. J. Eng. Res..

[12] Sadeghi S., Raki G., Amini A., Mengelizadeh N., Amin M.M., Hashemi M (2018). Study of the effectiveness of the third generation polyamideamine and polypropylene imine dendrimers in removal of reactive blue 19 dye from aqueous solutions TT. EHEMJ.

[13] Amirmahani N., Mahdizadeh H., Malakootian M., Pardakhty A., Mahmoodi N.O (2020). Evaluating nanoparticles decorated on Fe3O4@SiO2-schiff base (Fe3O4@SiO2-APTMS-HBA) in adsorption of ciprofloxacin from aqueous environments. J. Inorg. Organomet. Polym. Mater..

[14] Malakootian M., Yazdanpanah G., Poorjahanshahi M (2017). A comparison of the effectiveness of electrocoagulation to coagulation processes using ferric chloride for the removal of cadmium from aqueous solution. Desalin. Water Treat..

[15] Suresh A., Sathish S., Narendrakumar G (2019). Electrocoagulation of azo dye containing synthetic wastewater using monopolar iron electrodes and the characterization of the sludge. Water Pract. Technol..

[16] Yaghmaeian K., Silva Martinez S., Hoseini M., Amiri H (2016). Optimization of As(III) removal in hard water by electrocoagulation using central composite design with response surface methodology. Desalin. Water Treat..

[17] Malakootian M., Shahesmaeili A., Faraji M., Amiri H., Silva Martinez S (2020). Advanced oxidation processes for the removal of organophosphorus pesticides in aqueous matrices: a systematic review and meta-analysis. Process Saf. Environ. Prot..

[18] Meerbergen K., Crauwels S., Willems K.A., Dewil R., Van Impe J., Appels L. (2017). Decolorization of reactive azo dyes using a sequential chemical and activated sludge treatment. J. Biosci. Bioeng..

[19] Malakootian M., Smith J., Gharaghani M., Mahdizadeh H., Nasiri A., Yazdanpanah G (2020). Decoloration of textile Acid Red 18 dye by hybrid UV/COP advanced oxidation process using ZnO as a catalyst immobilized on a stone surface. Desalin. Water Treat..

[20] Tamaddon F., Mosslemin M.H., Asadipour A., Gharaghani M.A., Nasiri A (2020). Microwave-assisted preparation of ZnFe2O4@methyl cellulose as a new nano-biomagnetic photocatalyst for photodegradation of metronidazole. Int. J. Biol. Macromol..

[21] Malakootian M., Nasiri A., Khatami M., Mahdizadeh H., Karimi P., Ahmadian M. (2019). Experimental data on the removal of phenol by electro-H2O2 in presence of UV with response surface methodology. MethodsX.

[22] Malakootian M., Khatami M., Mahdizadeh H., Nasiri A., Amiri Gharaghani M (2020). A study on the photocatalytic degradation of p-nitroaniline on glass plates by thermo-immobilized ZnO nanoparticle. Inorg. Nano-Metal Chem..

[23] Malakootian M., Nasiri A., Amiri Gharaghani M (2020). Photocatalytic degradation of ciprofloxacin antibiotic by TiO2 nanoparticles immobilized on a glass plate. Chem. Eng. Commun..

[24] Nasiri A., Tamaddon F., Mosslemin M.H., Gharaghani M.A., Asadipour A (2019). New magnetic nanobiocomposite CoFe2O4@methylcellulose: facile synthesis, characterization, and photocatalytic degradation of metronidazole. J. Mater. Sci. Mater. Electron..

[25] Tamaddon F., Nasiri A., Yazdanpanah G (2020). Photocatalytic degradation of ciprofloxacin using CuFe2O4@methyl cellulose based magnetic nanobiocomposite. MethodsX.

[26] Malakootian M., Nasiri A., Alibeigi A.N., Mahdizadeh H., Gharaghani M.A (2019). Synthesis and stabilization of ZnO nanoparticles on a glass plate to study the removal efficiency of acid red 18 by hybrid advanced oxidation process (Ultraviolet/ZnO/ultrasonic). Desalin. Water Treat..

[27] Malakootian M., Nasiri A., Asadipour A., Kargar E (2019). Facile and green synthesis of ZnFe2O4@CMC as a new magnetic nanophotocatalyst for ciprofloxacin degradation from aqueous media. Process Saf. Environ. Prot..

[28] Malakootian M., Olama N., Malakootian M., Nasiri A (2019). Photocatalytic degradation of metronidazole from aquatic solution by TiO2-doped Fe3+ nano-photocatalyst. Int. J. Environ. Sci. Technol..

[29] Nasiri A., Tamaddon F., Mosslemin M.H., Faraji M (2019). A microwave assisted method to synthesize nanoCoFe2O4@methyl cellulose as a novel metal-organic framework for antibiotic degradation. MethodsX.

[30] Malakootian M., Nasiri A., Asadipour A., Faraji M., Kargar E (2019). A facile and green method for synthesis of ZnFe2O4@CMC as a new magnetic nanophotocatalyst for ciprofloxacin removal from aqueous media. MethodsX.

[31] Chavoshani A., Amin M.M., Asgari G., Seidmohammadi A., Hashemi M (2018). Advanced Oxidation Processes for Wastewater Treatment: emerging Green Chemical Technology.

[32] Malakootian M., Nasiri A., Heidari M.R (2019). Removal of phenol from steel plant wastewater in three dimensional electrochemical (TDE) process using CoFe2O4@AC/H2O2. Z. Phys. Chem..

[33] Malakootian M., Kannan K., Gharaghani M.A., Dehdarirad A., Nasiri A., Shahamat Y.D. (2019). Removal of metronidazole from wastewater by Fe/charcoal micro electrolysis fluidized bed reactor. J. Environ. Chem. Eng..

[34] Malakootian M., Mahdizadeh H., Khavari M., Nasiri A., Gharaghani M.A., Khatami M. (2020). Efficiency of novel Fe/charcoal/ultrasonic micro-electrolysis strategy in the removal of acid red 18 from aqueous solutions. J. Environ. Chem. Eng..

[35] Kornaros M., Lyberatos G. (2006). Biological treatment of wastewaters from a dye manufacturing company using a trickling filter. J. Hazard Mater..

[36] Sutherland A.J., Ruiz-Caldas M.-.X., de Lannoy C.-.F (2020). Electro-catalytic microfiltration membranes electrochemically degrade azo dyes in solution. J. Memb. Sci..

[37] Ahari S.M., Yangejeh R.J., Mahvi A.H., Shahamat Y.D., Takdastan A (2019). A new method for the removal of ammonium from drinking water using hybrid method of modified zeolites/catalytic ozonation. Desalin. Water Treat..

[38] Amirmahani N., Mahmoodi N.O., Bahramnejad M., Seyedi N (2020). Recent developments of metallic nanoparticles and their catalytic activity in organic reactions. J. Chin. Chem. Soc..

[39] Amirmahani N., Mahmoodi N.O., Malakootian M., Pardakhty A., Seyedi N (2020). Pd nanoparticles supported on Fe3O4@SiO2-Schiff base as an efficient magnetically recoverable nanocatalyst for Suzuki–Miyaura coupling reaction. Res. Chem. Intermed..

[40] Amirmahani N., Rashidi M., Mahmoodi N.O (2020). Synthetic application of gold complexes on magnetic supports. Appl. Organomet. Chem..

[41] Rajabizadeh K., Yazdanpanah G., Dowlatshahi S., Malakootian M (2019). Photooxidation process efficiency (UV/O3) for P-nitroaniline removal from aqueous solutions. Ozone Sci. Eng..

[42] Thomas R., Jenkins K., Landale B., Trigger G., Holsen T.M., Dore S. (2020). Evaluation of PFAS treatment technology: alkaline ozonation. Remediation.

[43] Normov D.A., Samarin G.N., Vasilyev A.N., Shevchenko A.A., Goldman R.B (2020). Proceedings of the 4th International Scientific and Technical Conference on Energy Systems, ICES.

[44] Schmitt A., Mendret J., Roustan M., Brosillon S (2020). Ozonation using hollow fiber contactor technology and its perspectives for micropollutants removal in water: a review. Sci. Total Environ..

[45] Wei X., Shao S., Ding X., Jiao W., Liu Y (2020). Degradation of phenol with heterogeneous catalytic ozonation enhanced by high gravity technology. J. Clean. Prod..

[46] Honarmandrad Z., Javid N., Malakootian M (2020). Efficiency of ozonation process with calcium peroxide in removing heavy metals (Pb, Cu, Zn, Ni, Cd) from aqueous solutions. SN Appl. Sci..

[47] Agudelo E.A., Cardona G S.A (2020). Selection of catalysts for use in a heterogeneous catalytic ozonation system. Ozone Sci. Eng..

[48] Zhiyong B., Yang Q., Wang J (2016). Catalytic ozonation of sulfamethazine using Ce0.1Fe0.9OOH as catalyst: mineralization and catalytic mechanisms. Chem. Eng. J..

[49] Zhao J., Cao J., Zhao Y., Zhang T., Zheng D., Li C (2020). Catalytic ozonation treatment of papermaking wastewater by Ag-doped NiFe2O4: performance and mechanism. J. Environ. Sci..

[50] Kim J., Lee J.E., Lee H.W., Jeon J.-.K., Song J., Jung S.-.C. (2020). Catalytic ozonation of toluene using Mn–M bimetallic HZSM-5 (M: fe, Cu, Ru, Ag) catalysts at room temperature. J. Hazard. Mater..

[51] Ma C., Jia S., Yuan P., He Z (2020). Catalytic ozonation of 2, 2′;-methylenebis (4-methyl-6-tert-butylphenol) over nano-Fe3O4@cow dung ash composites: optimization, toxicity, and degradation mechanisms. Environ. Pollut..

[52] Zeng W., Wang D., Luo Z., Yang J., Wu Z (2020). Phosphorus recovery from pig farm biogas slurry by the catalytic ozonation process with MgO as the catalyst and magnesium source. J. Clean. Prod..

[53] Zhang H., He Y., Lai L., Yao G., Lai B (2020). Catalytic ozonation of Bisphenol A in aqueous solution by Fe3O4–MnO2magnetic composites: performance, transformation pathways and mechanism. Sep. Purif. Technol..

[54] Li Y., Wu L., Wang Y., Ke P., Xu J., Guan B (2020). γ-Al2O3 doped with cerium to enhance electron transfer in catalytic ozonation of phenol. J. Water Process. Eng..

[55] Zhang X., Shen T., Ding Y., Tong S (2019). Graphite felt supported MgO catalytic ozonation of bisphenol A. Ozone Sci. Eng..

[56] Zhou L., Zhang S., Li Z., Liang X., Zhang Z., Liu R. (2020). Efficient degradation of phenol in aqueous solution by catalytic ozonation over MgO/AC. J. Water Process. Eng..

[57] Sobana N., Swaminathan M. (2007). The effect of operational parameters on the photocatalytic degradation of acid red 18 by ZnO. Sep. Purif. Technol..

[58] Zuorro A., Lavecchia R. (2013). Evaluation of UV/H2O2 advanced oxidation process (AOP) for the degradation of diazo dye Reactive Green 19 in aqueous solution. Desalin. Water Treat.

[59] Krishnakumar B., Swaminathan M. (2011). Influence of operational parameters on photocatalytic degradation of a genotoxic azo dye Acid Violet 7 in aqueous ZnO suspensions. Spectrochim. Acta – Part A Mol. Biomol. Spectrosc..

[60] Orge C.A., Faria J.L., Pereira M.F.R (2017). Photocatalytic ozonation of aniline with TiO2-carbon composite materials. J. Environ. Manag..

